# Effect of absenteeism on the performance of medical sciences students: gender differences

**DOI:** 10.1080/10872981.2021.1875531

**Published:** 2021-01-19

**Authors:** Abdulrahim Refdan Hakami

**Affiliations:** Department of Clinical Laboratory Sciences, College of Applied Medical Sciences, King Khalid University, Abha, Saudi Arabia

**Keywords:** Absenteeism, academic performance, sex, grade, self-learning

## Abstract

The effects of the learning environment on academic performance can be investigated according to a broad range of factors using a diversity of approaches. Many differences in academic performance have been associated with the sex of the student.

**Objectives**: This study aims to understand the impact of absenteeism on the final grades earned by full-time medical laboratory sciences undergraduate students and whether this is affected by sex. Academic performance was analyzed using students’ final grades from two consecutive semesters (January to April and September to December 2019). The differences between male (n = 43) and female (n = 72) students were evaluated by Pearson’s correlation. During the semester, all teaching and assessment methods were standardized across both course sections to avoid confounding effects derived from the teaching method. Academic performance was assessed both objectively (multiple-choice questions) and subjectively (short essay questions). The mean scores of male and female students during two semesters were significantly different (p = 0.0180). To correlate marks with absenteeism, the correlation coefficient (r) was negative, which indicates an inverse correlation between absence rate and scores. Interestingly, a statistically significant correlation between absenteeism and final grades was found in the male sample population (p = 0.0011 for the first semester; p = 0.0255 for the second semester) that was not observed for their female counterparts (p = 0.2041; p = 0.1537). The results indicate that academic performance among women is not solely dependent on class attendance but likely involves other factors such as self-learning, and group discussion. The mean scores of female medical sciences students were significantly higher than the male students for two consecutive semesters. Male overall scores seem to be conditional on the instructor’s explanation. This sex-based variation in academic performance revealed by taking absenteeism rate into account warrant further investigation.

## Background

Sex-based differences are currently highly researched and often controversial [1–3]. Academic achievement represents a considerable portion of this literature [4,5]. Many student-centered factors significantly impact academic outcomes including intelligence quotient (IQ) [6], learning differences [7], grit and perseverance [8], and teaching style (i.e. self-directed vs group study) [9–11]. Furthermore, the educational environment can be affected by personal and academic factors in students and teachers [12]; for example Rotthoff and colleagues examined the teachers’ perspectives [13]. Regarding the effect of absenteeism on academic performance, Latif Khan and colleagues study was based on lecture attendance. However, Gandhewar R and Vemulapalli K suggested few modifications of the study for more solid conclusion because some claims were overstated such as the statement that students who do not attend classes do not encourage positive learning environment [14,15]. The current study focused on the course learning outcomes to generate diverse questions that test different skills and these were correlated with absenteeism.

Within education assessment, absenteeism is a key variable that can affect students’ academic performance and its evaluation. Chronic absence, defined as an absence rate of 15% or more, can lead to a low level of math achievement and a reduced probability of graduation [16]. Qutub and colleagues’ study on absenteeism among Saudi medical students found that absenteeism impacted academic performance negatively. The study concluded that academic performance can be improved when students change their perspective toward attendance [17]. Another study on the effect of absenteeism on the academic performance of Saudi medical students surveyed 300 students using a cross-sectional questionnaire on 300 students [18]. Combining the survey results with the students’ grade point averages (GPAs) revealed that absenteeism significantly affected academic performance.

In a recent meta-analysis, Gubbels and colleagues reviewed the risk factors for school absenteeism [19]. The effect of absenteeism is most often investigated using student performance, namely test results and final grades, as the outcome measure [20,21]. However, very few studies address whether and how sex is involved in the observed outcome. Attitude is a confounding factor in these sex-based investigations. Gudaganavar and colleagues found no difference in the general attitudes of male and female students; although a significant sex-based difference was observed in study habits such as taking notes and preparing for exams [22]. Interestingly, better study habits were associated with higher academic achievement. The study habits of female students correlated significantly with their academic performance but this was not the case among male students. The relationships between sex, absenteeism, and academic performance in the medical sciences field warrants further exploration. Therefore, this study aimed to investigate whether male and female medical sciences students’ academic performance is differentially impacted by absenteeism.

## Methods

Preliminary investigations at the Clinical Laboratory Sciences Department, King Khalid University, indicated that female medical sciences students earn higher scores than their male counterparts. In the second semester of 2018, the scores from 37 courses showed that 17.52% of female students (n = 793) achieved an A+ while only 5.69% of male students (n = 474) obtained that mark. Therefore, this study was designed to understand this sex-based variation in academic performance using absenteeism as an independent variable. An advanced-level Clinical Virology course taken in the third-year was the focus of this study. The independent variable was the absence rate, and the outcome variable was the final grade. This observational study was conducted at King Khalid University, Saudi Arabia from January to December 2019. The sample population included third-year students enrolled in the mandatory Clinical Virology course. The course is taught in two sections every semester, which are separated by sex. The course coordinator taught the theory section for both sexes that constituted two-thirds of the overall grade. To mitigate subjective reporting bias, effects due to differences in scoring in both sections were controlled by using an assessment rubric specifically prepared by the course coordinator. The results from two semesters were included to increase the sample size, ensure reproducibility, and identify semester-based differences. The course is divided into basic science and clinical sections. The curriculum strictly adhered to the national accreditation standards.

At the beginning of every semester, a syllabus is distributed to the students that summarizes assessment tasks, topics, and the main textbook. There are four course learning outcomes from which the multiple-choice questions (MCQs) are generated. These outcomes were divided into three domains: Knowledge, skills, and competences. Two outcomes fall into competences domain. To match the questions with the learning outcomes, a matrix was used to map all MCQs and short-essay questions with the learning outcomes. The number of questions under each learning outcome was determined according to the relative weight of each outcome indicator. For example, the outcome of the knowledge domain was given a lower relative weight compared to skills and competence domain. The relative weight of the indicator is a value that represents the significance of each outcome taking into consideration their advanced-level as third-year students.

The final grade (out of 100) reflected student performance on a written assignment (summarize a book chapter) that was then presented orally; two midterm examinations; one practical evaluation; and one final examination. The two midterm examinations included the first basic science parts of the course, and the clinical part was included in the final examination. These tasks were graded using criteria sheets in table format. Each task was divided into criteria that should be covered in the answer. Short-essay questions were scored using a rubric with the essential themes necessary to earn a full mark for each question. For each 100-point test, 80% of the points were from MCQs and the remaining 20% from subjective short-essay questions. The MCQs were automatically scored using an SR-3500 HYBRID scanner (SEKONIC, Japan). The absence rate was calculated as a percentage of the overall class sessions. A 20% absentee rate was the maximum allowed to enter the final exam. Absence was recorded digitally by the course coordinator at every lecture.

## Data analysis

All data were normally distributed and analyzed using the GraphPad Software, version 8 (GraphPad Prism, San Diego, CA, USA). Differences between the mean final scores of male and female students were assessed using an unpaired t-test. For correlations between absenteeism, sex, and final score, linear regression and Pearson’s correlation analyses were used. The coefficient of determination (r^2^) was identified from a plot of the absence rate (X-axis) against the final score (Y-axis). A p-value of less than 0.05 was considered statistically significant.

## Ethics approval

The study was approved by the King Khalid University Ethics Committee (approval number: ECM#2020-183–(HAPO-06-B-001), and no identifying personal information (e.g. name, age) or other sensitive data were collected.

M = male students; F = female students

## Results

The study population was 115 third-year medical laboratory sciences students studying Clinical Virology, of which 37% were male and 63% female. Twenty-seven male students and 33 female students were included in the first semester (January to April 2019). Sixteen male students and 39 female students were enrolled in the second semester (September to December 2019).

Female students’ overall grades are higher than male students and this difference is statistically significant; p < 0.0001 for five semesters in the clinical virology course [23], and grades from their overall courses (Table 1). The same trend was found with another Bacteriology course in which the female section performed better than male students (Table 2). Here, data from two semesters supported this difference and was significant (p = 0.0184). This finding warrants further exploration to understand why female students outweigh male students by analyzing the possible effect of absenteeism on student success. Table 3 summarizes the average grade and average absence rate. A statistically significant difference was found between the average grades of male and female students; however, the absence rate was not significantly different between them.

**Table 1.** Students overall scores of all third-year courses

Section	Sex	(n)	Grades			
			A+	A	D+	D
2019 Semester I	M	84	1 (1.2%)	4 (4.7%)	42 (50%)	65 (77.3%)
	F	147	13 (8.8%)	21 (14.2%)	54 (36.7%)	47 (31.9%)
2019 Semester II	M	81	2 (2.4%)	8 (9.8%)	42 (51.8%)	47 (58%)
	F	144	14 (9.7%)	24 (16.6%)	27 (18.7%)	32 (22.2%)

**Table 2.** Third-year students overall scores and absence rates of the Bacteriology course

Section	Sex	(n)	Grades		Absence rate (average)
			A+	A	
2019 Semester I	M	9	0 (0%)	1 (11.1%)	4.58%
	F	19	3 (15.7%)	4 (21%)	16.66%
2019 Semester II	M	18	1 (5.5%)	1 (5.5%)	6.76%
	F	36	5 (13.8%)	4 (11.1%)	6.57%

**Table 3.** The average final grade and absence rate for both sexes in the clinical virology course

Variables	gender	Minimum	Maximum	Mean	SD	p-value
**Grade**	M (n = 43)	52.98	91.05	71.06	10.194	a0.0184
	F (n = 72)	59.59	93.08	77.13	7.495	
**Absence rate**	M (n = 43)	2.99	20.00	12.74	3.937	0.1652
	F (n = 72)	0.00	19.12	9.974	3.955	

a P-value < 0.05

**Figure 1.**
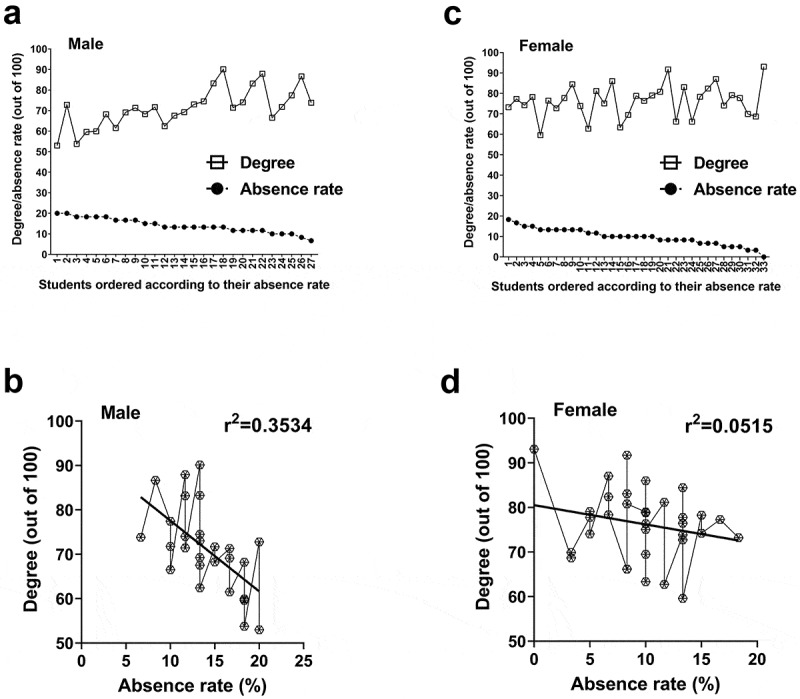
Correlation between absence rates and students’ final grades (January – April 2019 section). Male students exhibited a significant correlation between absence and grades (a and b) whereas a less dependent relationship between the two variables was found for female students (c and d). Male students: n = 27; Female students: n = 33

**Figure 2.**
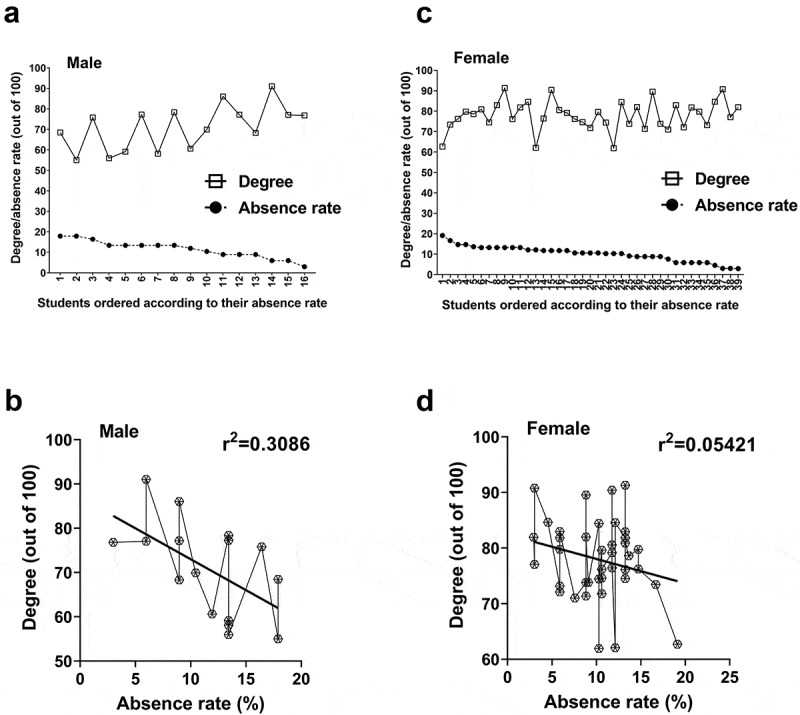
Correlation between absence rates and students’ final grades (September – December 2019 section). Male students displayed a significant correlation between absence and grades (a and b) whereas a less dependent relationship between the two variables was found for female students (c and d). Male students: n = 16; Female students: n = 39

Figures 1 and Figures 2 show the correlation between individual absences and grades. The correlation coefficients were negative (male: r = −0.5945 for the first semester/r = −0.5555 for the second semester; female: r = −0.2269/-0.2328). This indicates an inverse relationship between the absence rate and the final grade. Some outliers do not conform to this pattern. In the first semester, the student with no absences earned the highest overall grade (Figure 1(c)), but in the second semester, the student with the highest grade had an absence rate of 13%. To relate to the rest of the group, the average rate of absence (12.74%) is lower than 13% (Table 3).

To determine the nature of the relationship between absence and final grade these were plotted relative to each other and this plot was used to generate a best-fit regression line. From this line, male students’ correlations were r^2^ = 0.3534 and 0.3086 for the two consecutive semesters while r^2^ = 0.0515 and 0.05421 for their female counterparts (Figures 1 and Figures 2). The similarities within each sex across the semesters are striking and warrant in-depth discussion. In addition to r^2^, p-values were calculated using Pearson’s correlation analysis. A statistically significant correlation was found between the final grades versus absence rates for male students (p = 0.0011 for the first semester; p = 0.0255 for the second semester) but not female students (p = 0.2041 for the first semester; p = 0.1537 for the second semester).

## Discussion

Numerous factors impact the learning process and performance in the academic arena. The factors underlying sex-based differences in the study of medicine are poorly understood. Didactic teaching and the impact of active learning and interaction are all essential to teaching and learning, especially in the medical field. The finding that absenteeism correlated with academic performance in the male sample population aligns with observations that this group consistently earns lower average grades than their female counterparts, for whom statistically significant correlations between absence rate and final grade/academic performance were not observed. This provides some insight into why female’s average final grades are always higher than males’.

Academic achievement can be demonstrated and assessed in a diverse variety of forms (e.g. written, orally, active participation, technical skill). Concerning sex, Valli Jayanthi and colleagues used cumulative GPA to show that students’ grades are affected by extracurricular activities, nationality, sex and age [24]. As skill and technical ability represented a very small portion of the final score, these are likely not the basis for the sex-based difference observed in this investigation. Although a study assessed the spatial ability of medical students versus scores and observed sex-based variation [25].

To compare the current study with another finding that tested sex-based differences in academic performance, a study conducted at King Khalid University Hospital matched the findings of this study. The average GPA of female students at the Medical College was 3.94; while male students recorded 3.74. Another observation was the significant effect of absenteeism on academic performance, based on which they recommend implementing measures to reduce absenteeism [18]. In contrast, the results of the present study contradict those described by Kies and colleagues who found medical students’ performance did not vary according to sex [26]. They included a combination of online and paper-based examinations. The questions were mainly MCQs and the courses included neuroscience, biochemistry and medical statistics. These differences in method settings between Kies’ findings and this study could underlie the different results. They involved first-year medical students, but both studies investigated performance differences between male and female medical sciences students. One difference is that Kies examines the effect of written versus online examinations, while the current study focused on effect of absenteeism. Kies found no difference in performance while, in the present study, absenteeism affected the performance of male but not female students.

The negative correlation coefficients describing male and female sections alike support the common belief that absenteeism negatively affects academic outcomes. But further statistical analysis provided additional insight into the likelihood of this correlation by taking sex into account. Observing similar results across the two sections supports the preliminary data on sex-based differences in academic achievement.

The effect of absence on academic achievement was summarized by O’Dea and colleagues using teacher-assigned grades [27]. Here, subjective assessments of answers such as short-answer essay questions and MCQs were used to analyze the impact of absenteeism on the overall grade. Having found a sex-based similarity in the results across two consecutive semesters reflects consistency in teaching as explained in the context of accreditation standards, and supports that female students are more productive and better presented than their male counterparts in medical sciences with gaps in our awareness and understanding of the factors that drive this influence [28,29]. As the process of assessing the quality of teaching and learning for national accreditation is ongoing, the accreditation standards were applied including learning outcome evaluation using rubrics, performance indicators, and aligning the course learning outcomes with the program outcomes. Contradictory findings regarding assessment scores in medical education do exist [30,31], and the present research provides additional insight into this highly complex subject.

Latif Khan and colleagues used absenteeism as the principal outcome variable in their study of 404 male and female students. They found that the influence of sex disappeared when panel data predictors were incorporated and that class attendance correlated with final exam scores [21]. In an investigation of class attendance among second-year dermatology students, no statistically significant associations were found between performance on the final examination and attendance. The negative impact of absenteeism on academic performance was only observed in active learning sessions. However, their study was limited by a high mean score and small sample size [32]. They concluded that attending class did not enable dermatology students to perform better compared with watching online videos. Sex was also not thoroughly discussed even though the mean score among female students was higher than male students (87 versus 85 out of 100, respectively).

### Limitations

The present study has a few limitations. These data were from a single university and the sample size was relatively small. Additionally, course instructors’ assessments may differ from each other and student’s self-evaluation versus their assessment by instructors can vary [33]. These differences in assessment can muddle the analysis. This study did not investigate the students’ reasons for being absent. Hence, few medical reports were submitted by students to register excuses, and sickness was not the major cause of absence. Other studies have looked at this in details [34–36]. Another limitation is that the study design doesn’t allow drawing conclusions regarding causation or identifying actual interfering factors. On the other hand, a significant sex-based difference in academic achievement was observed in two separate semesters with limited absenteeism among female students. It is also difficult to differentiate between the effects of absence and motivation. For example, an excellent student might struggle to maintain a high level of attendance. Whether this observation is also true in other academic disciplines is an interesting topic for future study.

## Conclusions

By analyzing the final scores of medical sciences students from two academic semesters, a sex-based variation was observed in terms of academic performance that was inversely proportional to absenteeism in male students but separate from the absenteeism rate in female students. Sex was a confounding variable in the effect of absenteeism on academic performance. Other potential variables (e.g. specific behavioral and personal characteristics) were difficult to investigate in this context. Many interfering factors were proposed to either predict a causative factor or implicate other factors such as punctuality, IQ, learning style, motivation, perseverance, effort, and cognitive ability. Having data from two academic semesters describing one independent variable strengthens these findings. Taken together, absenteeism is a negative predictor of academic success for male but not female medical sciences students in Saudi Arabia.
